# Generation of macroscopic Schrödinger cat state in diamond mechanical resonator

**DOI:** 10.1038/srep37542

**Published:** 2016-11-23

**Authors:** Qizhe Hou, Wanli Yang, Changyong Chen, Zhangqi Yin

**Affiliations:** 1 National Laboratory of Solid State Microstructures and School of Physics, Nanjing University, Nanjing 210093, China; 2 State Key Laboratory of Magnetic Resonance and Atomic and Molecular Physics, Wuhan Institute of Physics and Mathematics, Chinese Academy of Sciences, Wuhan 430071, China; 3 Department of Physics, Shaoguan University, Shaoguan, Guangdong 512005, China; 4 Center for Quantum Information, Institute for Interdisciplinary Information Sciences, Tsinghua University, Beijing 100084, China

## Abstract

We propose a scheme to generate macroscopic Schrödinger cat state (SCS) in diamond mechanical resonator (DMR) via the dynamical strain-mediated coupling mechanism. In our model, the direct coupling between the nitrogen-vacancy (NV) center and lattice strain field enables coherent spin–phonon interactions in the quantum regime. Based on a cyclic Δ-type transition structure of the NV center constructed by combining the quantized mechanical strain field and a pair of external microwave fields, the populations of the different energy levels can be selectively transferred by controlling microwave fields, and the SCS can be created by adjusting the controllable parameters of the system. Furthermore, we demonstrate the nonclassicality of the mechanical SCS both in non-dissipative case and dissipative case. The experimental feasibility and challenge are justified using currently available technology.

Recently, the nitrogen-vacancy (NV) centers formed by a substitutional nitrogen atom and an adjacent lattice vacancy in diamond become the most promising solid-state platform for quantum information processing (QIP)^1^ and nanoscale sensors^2^, due to its easy controllability and fast manipulation^3^, as well as long coherence properties (electron spin ~1 *ms* and nuclear spin >1 *s*) in a wide temperature range, even at room-temperature^4^,^5^,^6^,^7^,^8^. Additionally, the NV center could couple to both optical and microwave fields simultaneously, which makes it possible be used as an excellent quantum interface between optical and solid-state systems^9^,^10^.

Meantime, the hybrid system consisting of a high-Q single-crystal diamond mechanical resonator (DMR) and embedded NV centers may provide a promising platform and open up a new perspective towards achieving quantum control and studying significant quantum optics or novel quantum phenomena^11^,^12^,^13^,^14^,^15^,^16^,^17^,^18^,^19^,^20^,^21^,^22^. In these systems, the embedded NV centers are highly susceptible to deformations of the surrounding lattice, where the strain field is robust against the dephasing or heating of the environment^12^,^13^. Applying this direct strain coupling mechanism, previous investigations have been focused on the mechanical spin driving^12^,^13^,^14^,^15^, enhancement of the coherence time of the NV center^16^, spin squeezing^17^, phonon cooling and lasing^18^.

On the other hand, based on the quantum superposition principle, the generation of the macroscopic quantum superposition state such as Schrödinger cat state (SCS) has attracted abundant attention because SCS plays an important role in the study of the foundations of quantum theory^23^,^24^,^25^,^26^,^27^,^28^,^29^,^30^,^31^, and brings many potentially applications, such as testing the wave function collapse^32^ and uncertainty relation^33^. Experimentally, the macroscopic SCS has been observed in various physical systems including superconducting qubit system^34^, ion trap^35^, cavity QED^36^, and linear optical system^37^. However, generating coherent states with macroscopically distinct amplitudes in the phase space is a difficult task in nanomechanical systems^38^,^39^,^40^, it remains a challenge to realize SCS in such systems.

In the present work, we introduce a scheme to generate macroscopic SCS in DMR via the dynamical strain-mediated coupling mechanism. In our model, the direct coupling between the lattice strain field and the NV center enables coherent spin–phonon interactions in the quantum regime, and the cyclic Δ-type transition structure of the NV center system could be constructed by combining the quantized mechanical strain field and a pair of external microwave fields, where one-photon and two-photon processes coexist. Using this cyclic population transfer, the population of the different energy levels of the NV center can be selectively transferred by controlling classical fields, and the SCS can be created by adjusting the controllable parameters of the system, such as the detuning between the NV center and the driving fields, along with their Rabi frequency. Here the mechanical SCS is described by the phase-space quasiprobability distribution, such as Husimi Q function and Winger function. Additionally, comparing with the non-dissipative case with the dissipative case through the superoperator method^41^,^42^, we find that the dissipation effect decreases the generation efficiency of SCS. Furthermore, we demonstrate the nonclassicality of the SCS in both non-dissipative case and dissipative case.

## Results

### System and Model

**Figure 1:**
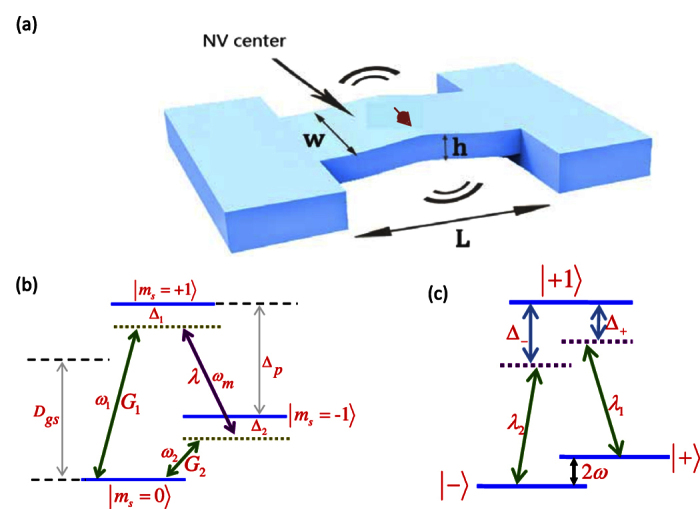
(**a**) Schematic of the hybrid system, where the NV electron spin (red row) is embedded in the DMR. *L, w*, and *h* 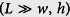 represent the length, width, and height of the DMR, respectively. (**b**) Levels of Δ-type transition ground-state electronic spin NV center, consisting of the levels of |0〉 and |±1〉, where, two classical microwave fields with frequencies *ω*_1_ and *ω*_2_ induce the transitions |0〉 ↔ |±1〉 with Rabi frequencies *G*_1_ and *G*_2_, respectively. In addition, the transition |+1〉 ↔ |−1〉 is coupled to the strain field (the frequency *ω*_*m*_) with the coupling strength *λ. D*_*gs*_ is the zero-field splitting between the state |0〉 and the nearly degenerated states |±1〉 which can be split by Δ_*p*_ in a magnetic field. Δ_1(2)_ are the detunings between the frequencies of the transition |0〉 ↔ |±1〉 and the frequencies of the microwave fields. (**c**) Level structure of the effective Λ-type transition structure. The transitions |±〉 ↔ |+1〉 are coupled to the displaced quantized fields with the coupling strengths *λ*_1_ and *λ*_2_, respectively. The energy spacing between energy levels |+〉 and |−〉 is 2*ω*.

As shown in Fig. 1(a), the system under consideration is a monolithic hybrid quantum device DMR including a diamond cantilever with an embedded NV center. The NV center consists of a substitutional nitrogen atom and a vacancy in an adjacent lattice site with *C*_3*v*_ symmetry^43^. It is negatively charged with two unpaired electrons located at the vacancy, and is treated as electronic spin *S* = 1. The ground state is spin triplet and labeled as |*m*_*s*_〉 with *m*_*s*_ = 0, ±1. The zero-field splitting between the spin sublevels |0〉 and |±1〉 is 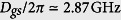, originating from the nonaveraged electronic spin-spin interactions^44^. In general, the quantum spin control of the NV center can be achieved with magnetic field^45^ or optical field^46^.

In our work, we mainly study individual NV center embedded in DMR, here the coherent strain driving of the NV center is based on the sensitive response of the spin to strain in the diamond host lattice. More specifically, when the DMR vibrates, it changes the local strain and induces a strain field at the position of the NV center. The strain field (behaved as an effective local electric field^44^) breaks the symmetry of the NV center and causes the energy shifts as well as a mixing of the states |+1〉 and |−1〉, which results in the direct coupling of the DMR and the transition |+1〉 ↔ |−1〉 of the NV center^43^,^44^. It offers a new approach to mechanical driving the NV spin. The Hamiltonian of the NV center coupling to the magnetic field and the strain field can be written as^17^,^44^,^47^ (in units of the Planck constant, i.e., *ħ* = 1, hereafter)


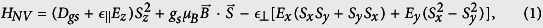


where 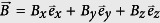 and 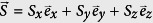 are the external magnetic field and spin-1 operator with *B*_*i*_ and *S*_*i*_ (*i* = *x, y, z*) the corresponding components, respectively. 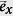, 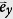, and 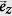 are unit vectors along the *x, y, z* direction. *ϵ*_‖(⊥)_ are the stress coupling constants along the direction parallel (perpendicular) to the NV symmetry axis (here we assume that the *z*-axis is aligned with the NV symmetry axis). 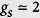, *μ*_*B*_ is the Bohr magneton. *E*_*i*_ (*i* = *x, y, z*) are the *i*-axis components of the strain field. In the *S*_*z*_ basis (*S*_*z*_|*m*_*s*_〉 = *m*_*s*_|*m*_*s*_〉), the spin operator can be expressed as 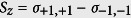, 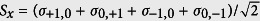, and   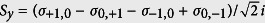 with *σ*_*j,k*_ = |*j*〉 〈*k*| (*j, k* = 0, ±1) the raising or lowering operators (for *j* ≠ *k*) and energy level populations (for *j* = *k*) of the NV center. Then, the Hamiltonian *H*_*NV*_ becomes


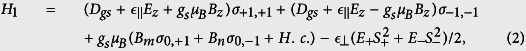


where *E*_±_ = *E*_*x*_ ± *iE*_*y*_, *S*_±_ = *S*_*x*_ ± *iS*_*y*_, 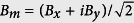, 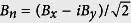, and the state |0〉 is set as the energy zero point.

The DMR can be treated as a harmonic oscillator with the Hamiltonian 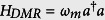, where *a* (*a*^†^) the annihilation (creation) operators of the quantized mode, and *ω*_*m*_ is the frequency of DMR. In addition, for small beam displacements, the perpendicular strain field can be quantized as *E*_+_ = *E*_0_*a* and *E*_−_ = *E*_0_*a*^†^ with *E*_0_ the strain on account of the zero point motion of the resonant mode^17^. The NV center in diamond is highly susceptible to deformations of the surrounding lattice, therefore, the vibration due to the ground mechanical mode of the nanoresonator changes the local strain where the NV center is located, and gives rising to an effective, strain-induced electric field *E*_0_, which results into the magnetically-forbidden transition |+1〉 ↔ |−1〉 with the related spin-phonon coupling strength *λ* = −*ϵ*_⊥_*E*_0_^43^,^44^. As a result, the cyclic Δ-type transition among all three spin levels of the NV center has been formed, as illustrated in Fig. 1b. The whole system can be written as 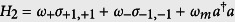 + 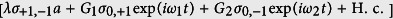, where H.c. represents the conjugate Hermitian, and 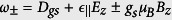. In the interacting picture, the Hamiltonian *H*_2_ changes into


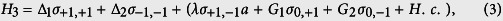


where Δ_1(2)_ = *ω*_+(−)_ − *ω*_1(2)_ represent the corresponding detunings between the transition frequencies *ω*_+(−)_ of the NV center and the frequencies of the classical microwave fields, respectively. Here the relation *ω*_1_ − *ω*_2_ = *ω*_*m*_ has been set.

### Generation of the SCS

In this section, we focus on how to generate the SCS via the dynamical strain-mediated coupling mechanism, based on the cyclic Δ-type transition structure.

To intuitively describe the main mechanism of how to create the SCS, we transform the cyclic Δ-type transition configuration in Fig. 1b into an effective Λ-type transition structure in Fig. 1c. Here the transitions |+1〉 ↔ |±〉 are induced by two displaced quantized phonon fields in Fig. 1c, which results from the quantized phonon field coupling to the transition |+1〉 ↔ |−1〉 in Fig. 1b, through an unitary transformation. Concretely speaking, the dressed states |±〉 have the following relation between the |−1〉 and |0〉 states as 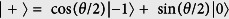 and 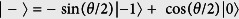 with the mixing angle *θ* = arctan[2*G*_2_/Δ_2_].

Based on this Λ-type transition structure in Fig. 1c, the effective Hamiltonian *H*_*eff*_ has the following form *H*_*eff*_ = *H*_11_ + *H*_+−_ with


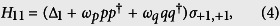


and


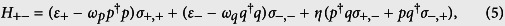


where *p* = *a* + *k*_1_ and *q* = *a* − *k*_2_ with *k*_1_ = *G*_1_ tan(*θ*/2)/*λ, k*_2_ = *G*_1_ cot(*θ*/2)/*λ. σ*_*j,k*_ = |*j*〉 〈*k*| (*j, k* = +1, ±). The other controllable parameters are *λ*_1_ = *λ* cos(*θ*/2), *λ*_2_ = *λ* sin(*θ*/2), 
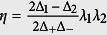
, 
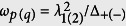
, 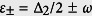, and Δ_±_ = Δ_1_ − *ε*_±_ with the dressed frequency 
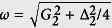
. The details of our deductions are presented in Section of Method.

In the large detuning case 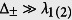, the upper state |+1〉 can be adiabatically eliminated by using the rotating wave approximation and the method of Fröhlich-Nakajima transformation (FNT)^48^. As a result, we obtain the Hamiltonian *H*_+−_ = *H*_+_*σ*_+,+_ + *H*_−_*σ*_−,−_ with


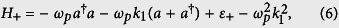


and


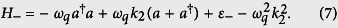


Note that the Hamiltonian *H*_+−_ can be employed to generate the SCS because both the Hamiltonian *H*_+_ and *H*_−_ also describe the forced harmonic oscillator.

Assuming that the whole system is initially in the state 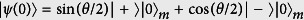, where the DMR is initially prepared in the vacuum state |0〉_*m*_, while the NV center is in the ground state 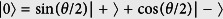
14. According to the unitary operator *U* = exp(−*iH*_+−_*t*), the whole system evolves into the time-dependent state


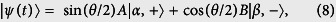


where 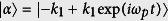 and 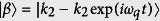 represent the coherent states of DMR. 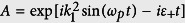 and 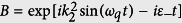 are the time-dependent global phases, and we denote the phase difference between |*α*〉 and |*β*〉 by *δ*_*AB*_. Note that the state 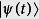 is an entangled cat state with different coherent states |*α*〉 and |*β*〉 (*α* ≠ *β*), and it is an entanglement between the mechanical phonon state and the NV center. If the readout result of the NV center is |0〉, the mechanical phonon state becomes 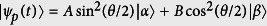, which is a standard mechanical SCS. In experiments, the relation between the population of |0〉 and the phase difference *δ*_*AB*_ has been used to directly detect the SCS 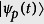. In our work, we will verify the existence of the mechanical SCS by calculating the overlap between these two coherent states |*α*〉 and |*β*〉 as


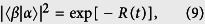



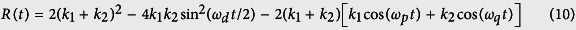


with *ω*_*d*_ = *ω*_*p*_ − *ω*_*q*_.

In the following, we use the quantum master equation to investigate the decoherence effect as


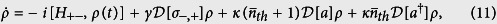


where 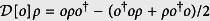 is the standard Lindblad superoperator that describes the dissipation effects. *γ* and *κ* represent the decay rate of the NV center and the DMR, respectively. 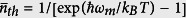 denotes the equilibrium phonon occupation number at temperature *T* with *k*_*B*_ the Boltzmann constant. In the basis {|+〉, |−〉}, using the superoperator method^41^, the analytical results can be obtained as


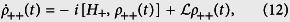



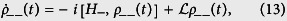



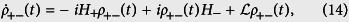



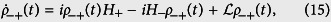


where 
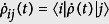
 (*i, j* = +, −) represent the matrix elements of the 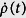, and the superoperator is 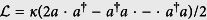. In our calculation, the operator disenable equation 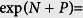 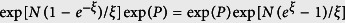 has been used, when 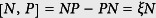. If the readout result of the NV center is |0〉, the density matrix with respect to the mechanical phonon state is


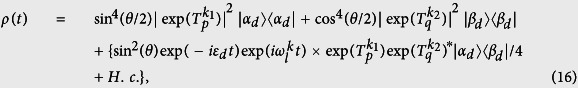


where, |*α*_*d*_〉 and |*β*_*d*_〉 represent the coherent states with 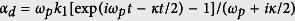 and 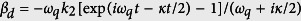. The other parameters are *ε*_*d*_ = *ε*_+_ − *ε*_−_, 
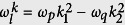
, and


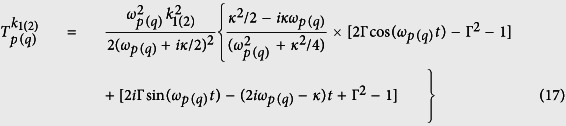


with dissipative factor 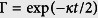. Using the similar method, we also calculate the overlap between theses two coherent states |*α*_*d*_〉 and |*β*_*d*_〉 in the dissipative case as 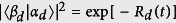 with


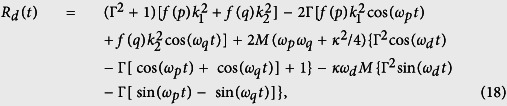


where 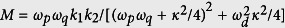 and 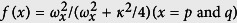.

**Figure 2:**
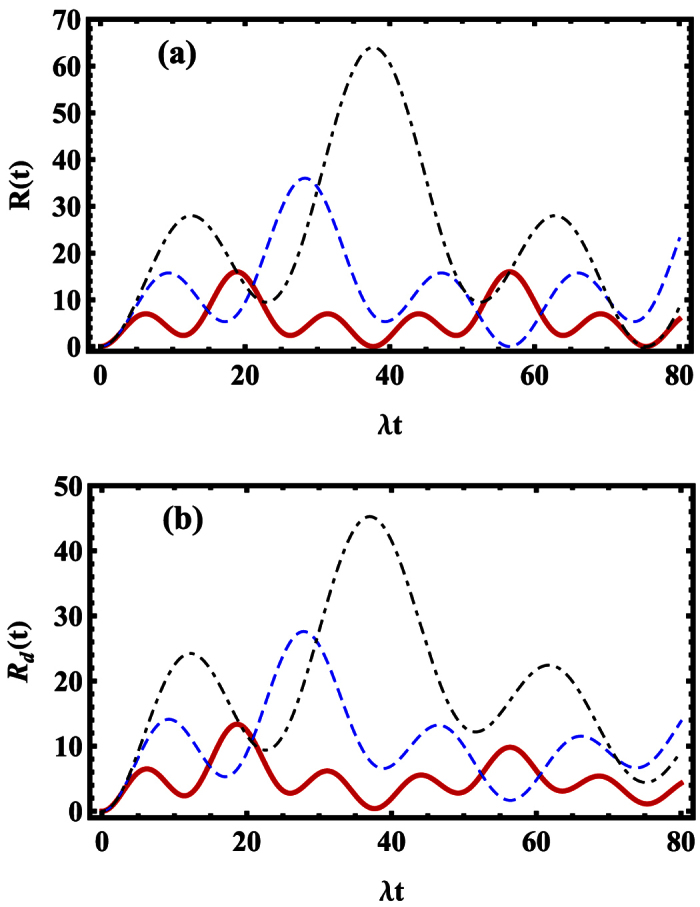
The relationship between the exponent *R(t*) (non-dissipative case, *κ* = 0) and *R*_*d*_(*t*) (dissipative case, *κ* = 0.02) and the time *t* is plotted in (**a**) and (**b**), respectively. The parameters are *λ* = 1, *G*_1_ = *G*_2_, Δ_1_ = 2*G*_2_, and *θ* = *π*/2. The red-solid line, blue-dashed line and black-dash-dotted line denote the case of *G*_2_ = 1, 1.5 and 2, respectively.

In Fig. 2, we plot the time evolution of *R(t*) (non-dissipative case) in Eq. (10) and *R*_*d*_(*t*) (dissipative case) in Eq. (18) under the different Rabi frequencies *G*_2_. In non-dissipative case, as shown in Fig. 2(a), the exponent *R(t*) of the overlap between coherent states changed periodically and symmetrically, and it has the identical maximum value in each period, which means that the overlap |〈*β*_*d*_|*α*_*d*_〉| becomes minimum at these times. Besides, we find that the larger the amount of Rabi frequency *G*_2_ is, the higher the maximal value of *R(t*) can reach, at the cost of the longer oscillation period. In dissipative case in Fig. 2(b), the symmetry and the periodicity on *R*_*d*_(*t*) is destroyed, and the maximum values of the exponent *R*_*d*_(*t*) decreases due to the dissipation effect. Combining this dissipative case with non-dissipative case, we come to the conclusion that the common feature that the small modulation overimposed on the larger modulation exists in both the non-dissipative and dissipative case, and the period of *R(t*) is partly determined by the Rabi frequency *G*_2_. Therefore the generation of SCS can be optimized by adjusting the Rabi frequency *G*_2_.

**Figure 3:**
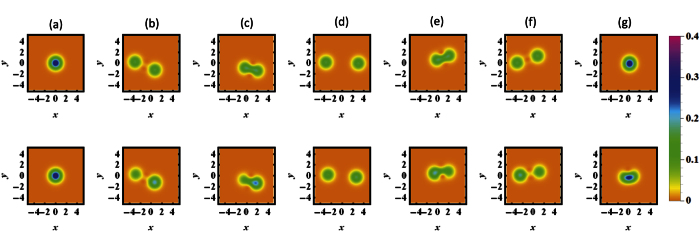
(**a**–**g**) Represent the Husimi Q function (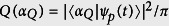 with *α*_*Q*_ = *x* + *iy* the arbitrary complex number and 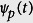 the mechanical phonon state) of SCS at the different times *t* = 0, 9, 17, 28, 39, 47, 56. The upper and lower panel represent the non-dissipative case (*κ* = 0) and dissipative case (*κ* = 0.02), respectively. The other parameters are the same as the Fig. 2(a).

To visualize the time evolution of the mechanical phonon state, we firstly calculate the phase-space quasiprobability distribution using the Husimi Q function^49^,^50^ spanned by the dimensionless field quadratures Re(*α*_*Q*_) and Im(*α*_*Q*_). The Husimi Q function is defined as 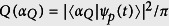 with *α*_*Q*_ = *x* + *iy* the arbitrary complex number and 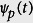 the mechanical phonon state. In Fig. 3, we plot the Husimi Q function of DMR phonon state at different times as *t* = 0, 9, 17, 28, 39, 47, 56, which correspond to the extreme time point of the exponent *R(t*) (*G*_2_ = 1.5) in the first period in Fig. 2(a). In the upper panel of Fig. 3 (the non-dissipative case with *κ* = 0), one can find that the DMR is initially prepared in the vacuum state, then gradually changed into the SCS, and finally evolve back into the vacuum state. In the presence of dissipation effect, i.e., *κ* = 0.02, as shown in the lower panel of the Fig. 3, the SCS can also be obtained with high-fidelity because the dissipation effect brings slight influence.

**Figure 4:**
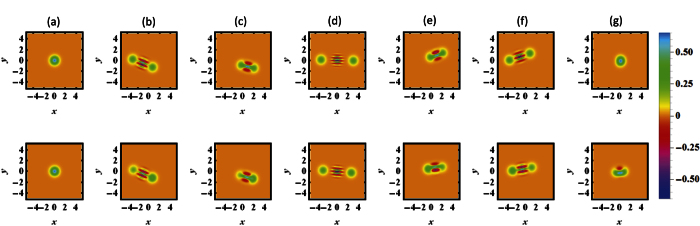
(**a**–**g**) Represent the Wigner function (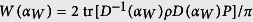 with the displacement operator 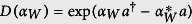 (*α*_*W*_ = *x* + *iy* is the arbitrary complex number), the density operator *ρ* of the mechanical phonon state and the parity operator *P* = exp(*iπa*^†^*a*)) of SCS at the different times *t* = 0, 9, 17, 28, 39, 47, 56. The upper and lower panel represent the non-dissipative case (*κ* = 0) and dissipative case (*κ* = 0.02), respectively. The other parameters are the same as the Fig. 2(a).

To show the interference fringes and nonclassicality of the mechanical SCS, the phase-space quasiprobability distribution using the Wigner function was also calculated in the phase space^51^, as shown in Fig. 4. In general, the quantum state could be judged to be nonclassical by checking that the Wigner function is negative in phase space. The Wigner function has the relation with the density operator *ρ* of the quantum state in the Fock state representation as 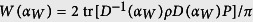 with the displacement operator 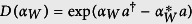 (*α*_*W*_ = *x* + *iy* is the arbitrary complex number) and the parity operator *P* = exp(*iπa*^†^*a*)^52^. In Fock space, we have *P*|*n*〉 = (−1)^*n*^ |*n*〉. Similar to the Husimi Q function, we plot the Wigner function on the SCS at the different times under the non-dissipative case (upper panel of the Fig. 4) and dissipative case (lower panel of the Fig. 4). One can find that the interference fringes appear under these two different cases, and it verifies the quantum features of the mechanical SCS of DMR due to the negative values of the Wigner function. Note that several experimental schemes^53^,^54^ have been proposed to measure the Winger function with respect to the vibrational cat state.

**Figure 5:**
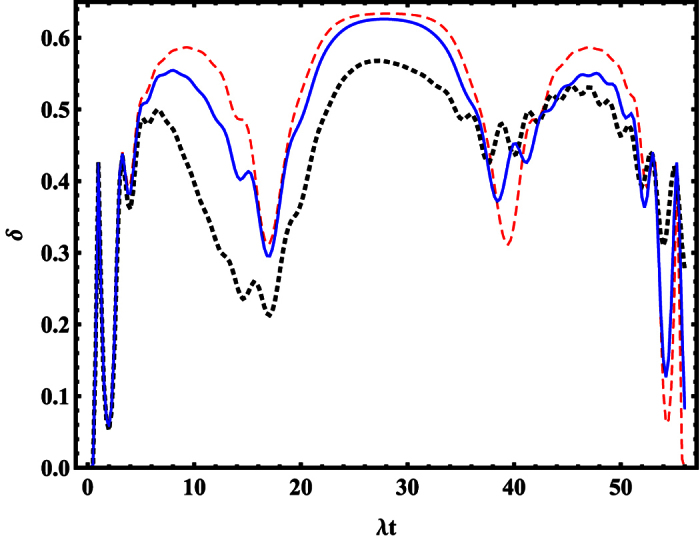
The negative volume *δ* of the Wigner function of SCS as a function of the time *t*. The red-dashed, blue-solid, and black-dashed lines represent the *κ* = 0, *κ* = 0.02, and *κ* = 0.05, respectively. The other parameters are the same as the Fig. 4.

To more describe quantitatively the quantum features of the mechanical SCS of DMR, we employ the negative volume *δ* of the Wigner function defined by Kenfack *et al*.^55^, as a quantitative measure of nonclassicality, where a non-zero value of *δ* indicates nonclassicality. The negative volume associated with a quantum state 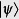 can be written as


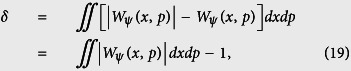


where


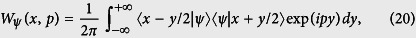


is the Wigner function of state 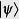 in the coherent state representation, which satisfies the normalization condition 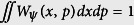. It means that the larger the value of the negative volume *δ* is, the stronger the classicality of the mechanical phonon state is. In Fig. 5, the negative volume *δ* of Wigner function associated with the mechanical SCS is shown as a function of time *t*, where the non-zero value of *δ* verifies the nonclassicality of the SCS, and the dissipation effect decreases the nonclassicality of the SCS during most of the evolution process.

## Discussion

We briefly address the experimental feasibility of our scheme. In general, the strain field could be produced by means of the thin piezoelectric^11^,^12^,^13^,^14^,^15^,^16^,^17^ or piezomagnetic^56^,^57^ film grown on the surface of the diamond layer, where, the piezoelectric or piezomagnetic film behaves as a transducer that transforms the signal between the external electric or magnetic field signal and the lattice vibration. Applying a voltage (magnetic field) across the piezoelectric film (piezomagnetic film), the strain field formed into the DMR. It implies that the strain field can be well controlled by the external electric or magnetic field. For a doubly clamped diamond beam with dimensions 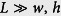, the coupling strength *λ* between the NV center and the DMR can be estimated by using the Euler-Bernoulli thin beam elasticity theory^58^. If the NV center is located near the surface of the beam, we have 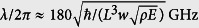 with *ρ* and *E* the mass density and the Young’s modulus of diamond, respectively. Assuming that the dimension of the DMR is on the scale of ~*μm*^17^ and the DMR’s vibrational frequency *ω*_*m*_ is about 0.1~10 GHz^18^, the coupling strength *λ* can be estimated as several kHz in our case. Besides, high-quality factor DMRs with *Q* = 10^5^ − 10^6^ have also been demonstrated in experiments^21^,^22^, therefore the mechanical damping rate *κ* = *ω*_*m*_/*Q* can be less than 1 kHz, which could be further decreased by reducing the DMR’s dimensions^59^,^60^ or improving the quality factor of DMR.

In addition, the preparation of the initial state in our scheme requires that the DMR is cool to the quantized ground state and the NV center is in the ground state, which can be realized in experiments. On one hand, several groups have demonstrated the mechanical resonator can be cooled to the quantum ground state^39^,^61^,^62^. For example, the experiment using conventional cryogenic refrigeration has demonstrated that the mechanical resonator can be cooled to its quantum ground state with the maximum phonon number 〈*n*_max_〉 < 0.07 and the probability >93%, through coupling the microwave-frequency mechanical oscillator to a superconducting phase qubit^39^. Additionally, recent work^62^ has realized an approximately 10 MHz micromechanical oscillator cooling to the quantum ground state with the phonon occupation 0.34 ± 0.05 using the sideband cooling technique, where the mechanical resonator is parametrically coupled to a superconducting microwave resonant circuit. Very recently, the topical review^63^ summarized three theoretical phonon cooling protocols on the present DMR system in our model, established for both strain and magnetically mediated interactions between the orbital and spin degrees of freedom of NV center and DMR phonons. On the other hand, the ground state of the NV center is also derived experimentally^14^. In addition, the excellent coherence properties of NV center has been demonstrated in experiment with the spin relaxation time *T*_1_~100 seconds^64^ and the spin dephasing time *T*_2_ = 10 milliseconds^4^ at low temperature. Comparing with the decay of the DMR, the decay of the NV center is negligible. Furthermore, we emphasized that our work has the essential difference with the previous work^26^, where the coupling was achieved by means of a strong magnetic field gradient from a nearby magnetic tip, and the spatial quantum superposition state for the diamond’s center-of-mass oscillation was generated via the magnetic field gradient induced the NV center electron spin and the mechanical oscillation coupling. Here we use intrinsic strain coupling between mechanical oscillator and the NV center to generate the macroscopic quantum superposition states. Larger magnetic field gradient is not needed.

For more technical aspects, we notice that a hybrid device that integrating a microscale diamond beam with a single embedded NV center spin to a superconducting coplanar waveguide cavity was proposed^65^, where the diamond beam phonon can strongly couple to the cavity photon via a dielectric interaction through an ac electric field. Based on this method, quantum state transfer between the mechanical mode and the cavity can be realized. For instance, if we map the mechanical phonon state into the cavity field completely, the Wigner function of SCS can be constructed by quantum homodyne tomography via detecting the cavity photons^50^,^66^,^67^. In addition, several theoretical and experimental schemes have been proposed to detect the cat state in different physical systems, perhaps the most possible achievable measurement to our mechanical SCS is analogy of the experimental techniques applied in the system of trapped ion^68^,^69^, where the quantum mechanical interference between the localized wave packets of the motional states of ions can be directly measured by detecting the probability of the ion’s internal state.

In summary, we utilize the cyclic Δ-type transition structure in the DMR system to generate the nonclassical mechanical SCS through the dynamical strain-mediated coupling mechanism. Using this cyclic population transfer, the populations of the different energy levels of the NV center can be selectively transferred by controlling classical fields, and the SCS can be created by adjusting the controllable parameters of the system. Our scheme also provides another route for building a distributed QIP architecture, where each DMR unit acts as a quantum node, and quantum information is transferred and processed in and between the NV centers at spatially different DMR. It may be a significant step toward the future full-scale quantum-information processor based on increasingly developed nanoscale solid-state technology.

## Method

### The deduction of the Hamiltonian *H*_+−_ (**Eqs (6 and 7)**) using the FNT method

Firstly, we consider the sub-Hamiltonian 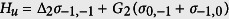 containing the two lower states |0〉 and |−1〉 of the NV center. It is easy to derive its eigenstates as 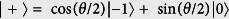 and 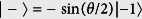 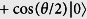 and the corresponding eigenvalues 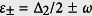. The mixing angle is *θ* = arctan[2*G*_2_/Δ_2_] and the dressed frequency is 
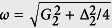
.

Thus, we can rewritten the total Hamiltonian of the system *H* as *H* = *H*_*f*_ + *H*_*i*_ with


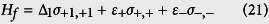


and


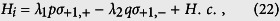


where *p* = *a* + *k*_1_ and *q* = *a* − *k*_2_ can be treated as the displaced boson operators. The other four controllable parameters are *λ*_1_ = *λ* cos(*θ*/2), *λ*_2_ = *λ* sin(*θ*/2), *k*_1_ = *G*_1_ tan(*θ*/2)/*λ*, and *k*_2_ = *G*_1_ cot(*θ*/2)/*λ*, respectively. Therefore, the effective Λ-like three-level transition of NV center has been established, as shown in Fig. 1c. The transition |+1〉 ↔ |±〉 are coupled to the displaced quantized phonon field with the operators *p* and *q*, respectively, and the corresponding coupling strengths are *λ*_1_ and *λ*_2_. As a result, the quantized phonon field coupling to the transition |+1〉 ↔ |−1〉 has been transformed into two displaced quantized phonon field coupling two transition |+1〉 ↔ |±〉 by this unitary transformation.

In order to better understand the mechanism to prepare the nonclassical mechanical SCS, in the large detuning limit 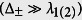, we can separate the upper state |+1〉 from the two lower states |±〉 and adiabatically eliminate the transitions |+1〉 ↔ |±〉 by using the FNT method^48^. We consider the total Hamiltonian *H* consisting of the free Hamiltonian *H*_*f*_ and the interaction Hamiltonian *H*_*i*_, and use the general perturbation theory to treat the interaction part *H*_*i*_ as a perturbation term comparing with the free part *H*_*f*_. Then, an unitary transformation is used to the Hamiltonian *H*, and derived an effective Hamiltonian *H*_*eff*_ as 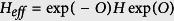 with *O* an anti-Hermitian operator. Further, by using the Baker-Campbell-Hausdorff formula^70^, the effective Hamiltonian *H*_*eff*_ could be simplified to


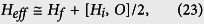


where the condition *H*_*i*_ + [*H*_*f*_, *O*] = 0 has been used, which determines the form of anti-Hermitian operator *O*.

In general, the eigenvectors {|*i*〉 = |+1〉, |±〉} (corresponding to the eigenvalues *E*_*i*_ = Δ_1_, *ε*_±_) for the free Hamiltonian *H*_*f*_ are known. Taking the matrix elements of the equation *H*_*i*_ + [*H*_*f*_, *O*] = 0 on the basis |*i*〉, we can obtain 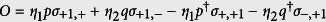 with 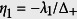, 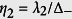, and Δ_±_ = Δ_1_ − *ε*_±_.

Then, substituting the expression of *H*_*f*_, *H*_*i*_, and *O* into the Eq. (23), the effective Hamiltonian *H*_*eff*_ can be rewritten as *H*_*eff*_ = *H*_11_ + *H*_+−_ with


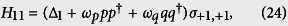


and


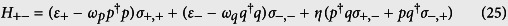


with the parameters 
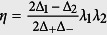
 and 
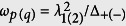
.

By substituting the expressions *p* = *a* + *k*_1_ and *q* = *a* − *k*_2_ into the Hamiltonian *H*_11_, we can obtain 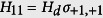 with 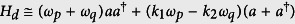 describing a typical forced harmonic oscillator.

In addition, using the rotating wave approximation, we write the Hamiltonian *H*_+−_ as *H*_+−_ = *H*_+_*σ*_+,+_ + *H*_−_*σ*_−,−_ with


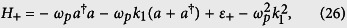


and


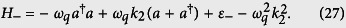


## Additional Information

**How to cite this article**: Hou, Q. *et al*. Generation of macroscopic Schrödinger cat state in diamond mechanical resonator. *Sci. Rep.* **6**, 37542; doi: 10.1038/srep37542 (2016).

**Publisher's note:** Springer Nature remains neutral with regard to jurisdictional claims in published maps and institutional affiliations.
